# Intensely clustered outbreak of visceral leishmaniasis (kala-azar) in a setting of seasonal migration in a village of Bihar, India

**DOI:** 10.1186/s12879-019-4719-3

**Published:** 2020-01-06

**Authors:** Arvind Kumar, Suman Saurabh, Sarosh Jamil, Vijay Kumar

**Affiliations:** 1 Vector Borne Diseases Control officer – Sheikhpura district, Health Department, Government of Bihar, India. Currently, Chief Medical Officer – Arwal district, Health Department, Government of Bihar, Sheikhpura, India; 2 Zonal Coordinator - Neglected Tropical Diseases, Muzaffarpur, World Health Organization - India. Currently, Assistant Professor, Department of Community and Family Medicine, All India Institute of Medical Sciences (AIIMS) - Jodhpur, Jodhpur, Rajasthan 342005 India; 3 Zonal Coordinator - Neglected Tropical Diseases, Bhagalpur, World Health Organization - India. Currently, State Coordinator – Neglected Tropical Diseases, World Health Organization – India, Raipur, Chhattisgarh India; 4 Consultant and Ex-Scientist E, Department of Vector Biology & Control, Rajendra Memorial Research Institute of Medical Sciences (Indian Council of Medical Research), Patna, India

**Keywords:** Kala-azar, Visceral leishmaniasis, Outbreak, Epidemic, Migration, Indoor residual spraying, India

## Abstract

**Background:**

A visceral leishmaniasis outbreak was reported from a village in a low-endemic district of Bihar, India.

**Methods:**

Outbreak investigation with house-to-house search and rapid test of kala-azar suspects and contacts was carried out. Sandfly collection and cone bio-assay was done as part of entomological study.

**Results:**

A spatially and temporally clustered kala-azar outbreak was found at Kosra village in Sheikhpura district with 70 cases reported till December 2018. Delay of more than a year was found between diagnosis and treatment of the index case. The southern hamlet with socio-economically disadvantaged migrant population was several times more affected than rest of the village (attack rate of 19.0% vs 0.5% respectively, OR_MH_ = 39.2, 95% CI 18.2–84.4). The median durations between onset of fever to first contact with any health services, onset to kala-azar diagnosis, diagnosis to treatment were 10 days (IQR 4–18), 30 days (IQR 17–73) and 1 day (IQR 0.5 to 3), respectively, for 50 kala-azar cases assessed till June 2017. Three-fourths of these kala-azar cases had out-of-pocket medical expenditure for their condition. Known risk factors for kala-azar such as illiteracy, poverty, belonging to socially disadvantaged community, migration, residing in *kutcha* houses, sleeping in rooms with unplastered walls and non-use of mosquito nets were present in majority of these cases. Only half the dwellings of the kala-azar cases were fully sprayed. Fully gravid female *P. argentipes* collected post indoor residual spraying (IRS) and low sandfly mortality on cone-bioassay indicated poor effectiveness of vector control.

**Conclusions:**

There is need to focus on low-endemic areas of kala-azar. The elimination programme should implement a routine framework for kala-azar outbreak response. Complete case-finding, use of quality-compliant insecticide and coverage of all sprayable surfaces in IRS could help interrupt transmission during outbreaks.

## Background

Visceral leishmaniasis (kala-azar) is a disease caused by *Leishmania donovani*, a protozoal parasite and is transmitted to humans through the bite of *Phlebotomus argentipes* sandfly [1]. Owing to presence of only human reservoir and high cure rates of kala-azar in the Indian sub-continent (Bangladesh, India and Nepal), kala-azar has been targeted for elimination as a public health problem [2]. India has already achieved a remarkable ten-fold reduction in annual kala-azar cases from 2007 to 2018 [3, 4]. A target of reduction of annual incidence below 1 per 10,000 population at sub-district (block) level by 2020 has been adopted for elimination in India [2, 5].

Kala-azar has a long history of outbreaks with the earliest recorded outbreak in 1824–25 in Jessore district of present day Bangladesh [6, 7]. It also shows a cyclical trend every 15–20 years [6, 7]. Outbreaks of kala-azar in previously non-endemic areas have also been reported [8–10]. Therefore, to sustainably achieve the goal of elimination, attention is also needed on blocks with kala-azar incidence below the elimination target. Consequently, Accelerated Plan for Kala-azar Elimination was launched in February 2017 emphasizing enhanced surveillance in borderline and low-endemic blocks with annual incidence of 0.8–1 and less than 0.8 per 10,000 population, respectively [11].

**Fig. 1:**
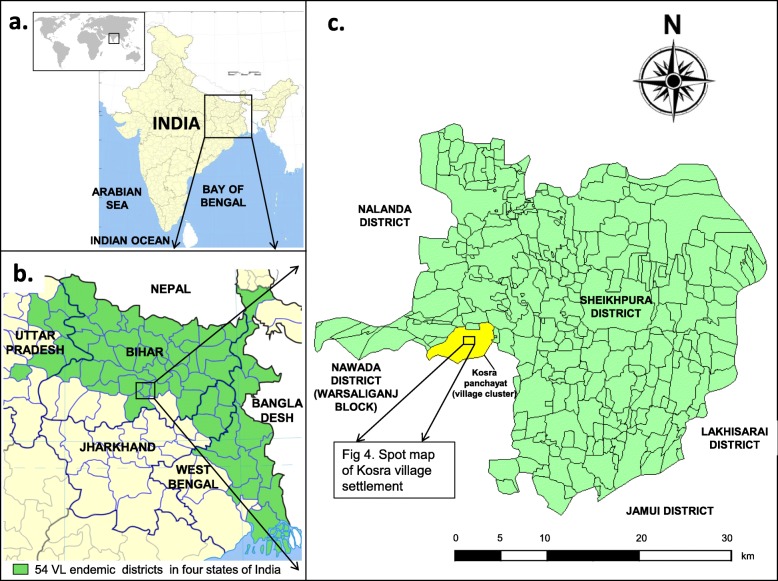
Location of the outbreak in Sheikhpura, Bihar, India – **a**) location of kala-azar endemic area in the eastern part of India **b**) location of Sheikhpura district among the kala-azar endemic districts in India **c**) location of the affected village within Sheikhpura district. *(Modified from source file -* https://commons.wikimedia.org/wiki/File:India_districts_map.svg*, Creative Commons Attribution-Share Alike 4.0 International license)*

A kala-azar outbreak was reported by the Bihar state government in March 2017 at Kosra village in Sheikhpura district. This district lies in the fringe of the kala-azar endemic zone in India (Fig. 1). With annual kala-azar incidence of 0.19 per 10,000 population in 2016, it ranked 22nd among the 33 endemic districts of Bihar. The affected village is in Sheikhpura Sadar block,12 km from the district headquarters. It borders Sekhopur Sarai block of Sheikhpura district and Warsaliganj block of Nawada district (Fig. 1).

**Fig. 2:**
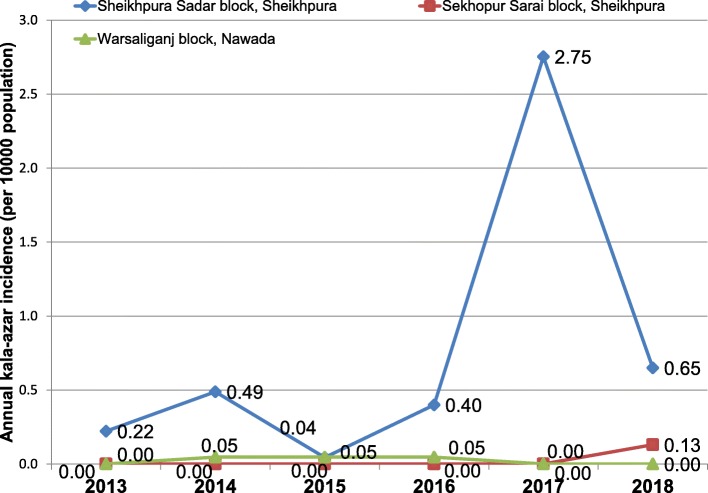
Reporting of kala-azar cases from Sheikhpura sadar block, Sheikhpura Warsaliganj block, Nawada adjacent to Kosra village (2013–2018) Data source: District Vector Borne Disease Control Office, Sheikhpura, Bihar, India.

Sheikhpura Sadar block and neighbouring Sekhopur Sarai block were low-endemic and had reported kala-azar cases sporadically from 2013 to 2016 (Fig 2). Neighbouring Warsaliganj block had not reported any kala-azar case during this period. As per the official line list available since January 2011, the affected village had not reported any case of kala-azar until the onset of the present outbreak. Kala-azar line list prior to 2011 was not available in the district and sporadic cases used to directly get treated in the private sector and were not notified to the government.

## Methods

### Study design and planning

The outbreak response consisted of door-to-door case search, enumeration of the affected population and entomological assessment of effectiveness of vector control activities. An outbreak response team led by the District Vector Borne Disease Control Officer (DVBDCO) was constituted in June 2017. The team consisted of medical officers, a lab technician and frontline health workers associated with the primary health centre. Since the study was within the purview of programme evaluation and was part of an urgent outbreak response, it was exempt from prior institutional ethics committee approval as per national guidelines [12]. Existing pulse polio supplementary immunization micro-plan was used to divide the village into 6 clusters of 50–60 households each. A team of frontline health workers and medical officers visited the households and completely enumerated the area.

During the household visit, those with fever and contacts of known kala-azar cases were referred for rK39 rapid test (Kala-azar Detect™, InBios Inc., Seattle, USA) at the health sub-centre located in the village. Those with history of an earlier episode of kala-azar were excluded from rk39 test. Contacts were defined as those who resided in the same household as known kala-azar cases and their next-door neighbours. All rK39-positives with history of two or more weeks of fever were invariably referred to the government district hospital. Here they were confirmed as having kala-azar clinically. Those with two or more weeks of fever and finding of palpable spleen were treated with 10 mg / kg single dose liposomal amphotericin B (LAmB), as per programme guidelines [4]. HIV test was done for all patients. Other locally endemic differential diagnoses such as typhoid and malaria were excluded through laboratory tests ordered based on clinician’s judgement at district hospital. Since programme guidelines recommend parasitological confirmation only in specific situations at tertiary centres equipped with this facility, it was not attempted routinely for the patients referred to the district hospital [11].

A semi-structured questionnaire was developed for assessing kala-azar cases regarding awareness and access to treatment and preventive services. Known risk factors for kala-azar were also assessed among the cases. Interview of relatives of those who had died after prolonged fever was also done using the WHO Verbal Autopsy instrument 2016.

### Entomological study

Entomological study was conducted by scientists from Rajendra Memorial Research Institute of Medical Sciences (RMRIMS), Patna, India. Vector density was assessed through sandfly collection using mouth aspirator and CDC light trap in randomly selected households in July 2017, two days after focal indoor residual spraying (IRS) was conducted in the most affected southern hamlet. Species identification was done using taxonomic key described by Lewis [13]. Cone bio-assay was done on both mud and brick surfaces to provide estimate of insecticidal efficacy of IRS using WHO recommended procedures [14, 15].

### Data entry and analysis

**Fig. 3:**
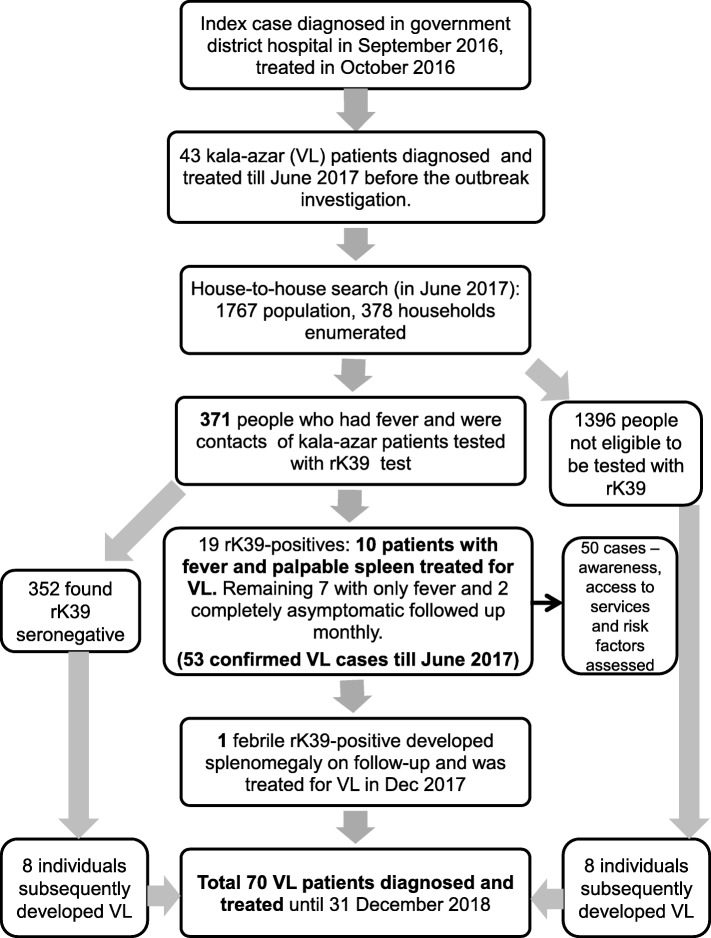
Flowchart showing evolution of the kala-azar outbreak until December 2018

Data was entered and cross-checked for errors. Age-wise, gender-wise and area of residence-wise attack rates were described using numerator of all kala-azar cases reported up to December 2018 (Fig. 3). The gender, age and month of fever onset and diagnosis of cases reported after the outbreak investigation in June 2017 were taken from the latest line list available in the district. The denominator was taken from the house to house enumeration conducted in June 2017.

**Fig. 4:**
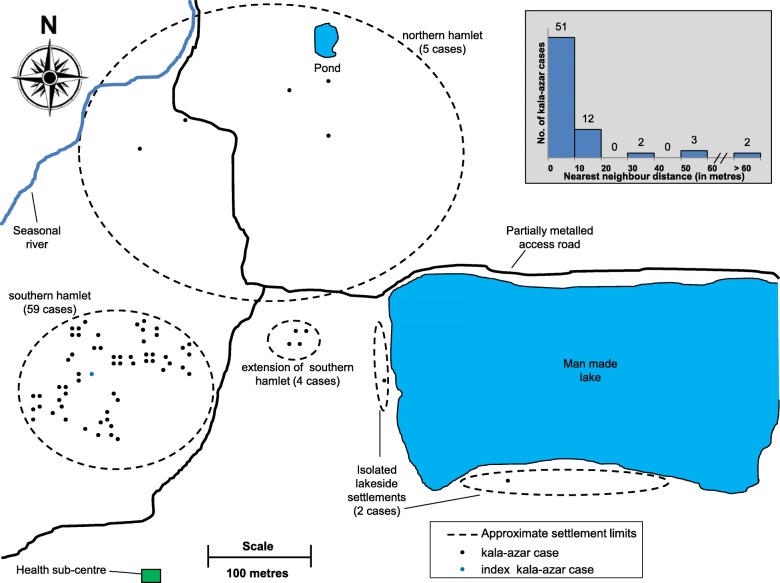
Spot map of the kala-azar cases in Kosra village and inset showing nearest neighbour distance of the cases (*n* = 70 cases). *(A computer-drawn schematic base map has been purposely inserted in place of satellite image so that spatial relationship is shown while individual cases’ location can’t be linked to specific houses. Satellite image of the village can be viewed separately at Earth Explorer-* https://earthexplorer.usgs.gov*by searching for ‘Kosra, Bihar, India’)*

Mantel-Haenszel method was used to statistically compare the attack rates of kala-azar for the most affected hamlet as compared to rest of the village, stratified by gender. The spot-map represented all kala-azar cases till December 2018 (Fig. 4). Nearest neighbour analysis of kala-azar cases was done with QGIS v3.2.3.

## Results

During this outbreak investigation, 371 people with fever and contacts of kala-azar patients were tested (Fig. 3). Nineteen individuals were found to be rK39-positive among whom 10 with two or more weeks of fever and palpable spleen were treated at the district hospital. None of the patients were found HIV positive. Out of the remaining 9 rk39-sero-positive individuals without palpable spleen, 7 had fever while 2 were completely asymptomatic (Fig. 3). The index case was found to have developed fever as early as June 2015 and was finally diagnosed with kala-azar at the district hospital in September 2016 and was treated with LAmB in October 2016.

The outbreak was ongoing with a size of 70 cases detected till December 2018 (Fig. 3). Additionally, 4 kala-azar cases have been reported recently in the village, one each in February, March, May and September 2019.

### Environmental characteristics

The wet season lasts from mid-June to September. The eco-environmental conditions found here are considered suitable for sandfly breeding [16, 17]. The settlement of Kosra village lies close to a man-made lake and a pond. A seasonal river is located to the west (Fig. 4). Water bodies and seasonal river banks have been geospatially associated with sandfly abundance [18].

### Description of population at risk and outbreak (70 kala-azar cases detected till December 2018)

The enumerated population of the Kosra village was 1767 with 52.2% males. A total of 378 households were enumerated. The village is divided into mainly two settlements (Fig. 4). The smaller hamlet in the south and its extension is entirely populated by the socio-economically disadvantaged *mushahar* community which is notified as a scheduled caste by the government. This hamlet has 72 households and a population of 341. The small isolated lakeside settlement comprises of 20 households and is inhabited by 110 people belonging to scheduled caste. The large northern hamlet has 286 households with a resident population of 1316, mainly comprising of population belonging to the relatively privileged castes.

**Fig. 5:**
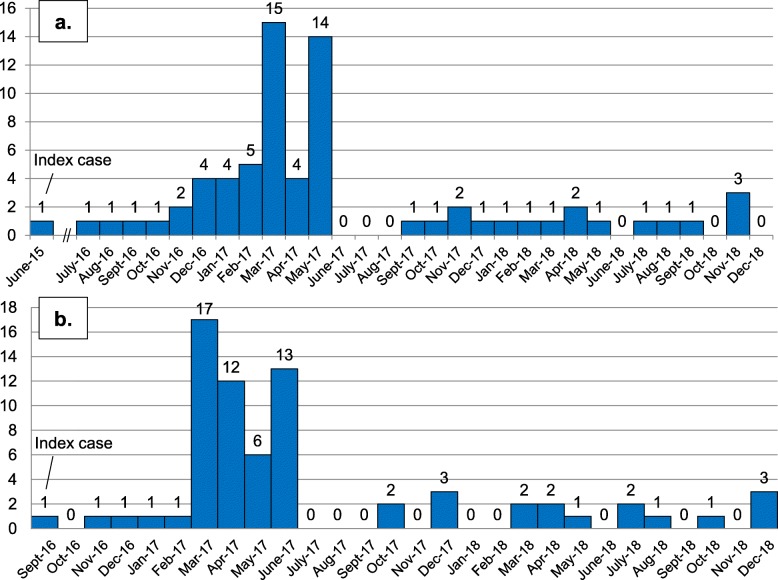
Time distribution of kala-azar cases (*n* = 70) at Kosra village until December 2018 – **a**) by month of developing fever and **b**) by month of being diagnosed for kala-azar

Most kala-azar cases were clustered in the southern hamlet around the index case whereas sporadic cases were found in the rest of the village (Fig. 4). Distances from one patient to another nearest patient had a left-skewed distribution (Median 7.35 m, interquartile range 6.39–10.36 m). More than two-thirds of kala-azar cases were residing within 10 m of another kala-azar case (Fig. 4). Around three-fourths of all kala-azar cases developed fever during a span of six months i.e. from November 2016 to May 2017. The upsurge in cases being diagnosed was seen only in March 2017, owing mainly to the early return of migrants after developing fever at their seasonal migration sites (Fig. 5). Thus a spatial and temporal clustering of kala-azar cases was found in the village.

Around one-third of the 70 kala-azar cases diagnosed till December 2018 belonged to 0–14-year age group. Age-group wise attack rates were not significantly different (Table 1). Around 61.4% of them were males. Male gender was not found to be a significant risk factor for being affected with kala-azar (Risk ratio = 1.44, 95% CI 0.90–2.31, *p* = 0.130).

**Table 1:** Area of residence and age-wise attack rates (incidence proportion) of kala-azar in Kosra village, stratified by gender (70 cases)

Characteristics	Male		Female		Total	
	No. of VL/ Population in group	Attack rate (%)	No. of VL/ population in group	Attack rate (%)	No. of VL/ population group	Attack rate (%)
Area of residence						
* Southern hamlet and its extension*	39/ 158	24.7	24/ 174	13.8	63/ 332	19.0
* Rest of the village*	4/ 759	0.5	3/ 667	0.4	7/ 1426	0.5
Age group*						
* 0–14*	14 / 365	3.8	9 / 320	2.8	23/ 685	3.4
* 15–29*	12 /199	6.0	9 / 232	3.9	21/ 431	4.9
* 30–44*	10 / 153	6.5	3 / 137	2.2	13/ 290	4.5
* 45–59*	3 / 98	4.1	4 / 90	4.4	7/ 188	3.7
* 60 +*	4 / 102	3.9	2 / 62	3.2	6/ 164	3.7
Total	43 / 917	4.7	27/841	3.2	70/ 1758	4.0

Since gender details of 9 enumerated individuals were missing, denominator is 1758 instead of 1767. * For age-group, Chi-square = 1.86, df = 4, *p* = 0.762. VL – Visceral leishmaniasis (kala-azar*)*

Kala-azar attack-rate in the southern hamlet and its extension was several times higher than in the rest of the village (19.0% vs 0.5%). Mantel-Haenszel adjusted risk ratio for being affected by kala-azar for residents of southern hamlet was 39.2 (95% CI 18.2–84.4, *p* < 0.0001) as compared to rest of the village, while taking gender as stratifying variable.

### Out of pocket expenditure, delay in diagnosis and treatment, access to services and awareness of the cases

Fifty kala-azar patients who could be interviewed out of the 53 reported till June 2017 were assessed for out of pocket expenditure, delay in diagnosis and treatment, access to services and awareness (Table 2). The median durations between onset of fever to first contact with any health services, onset to diagnosis, diagnosis to treatment were 10 days (IQR 4–18), 30 days (IQR 17–73) and 1 day (IQR 0.5 to 3) respectively.

**Table 2:** Health seeking behaviour, access to diagnosis, treatment and preventive services and risk factors for kala-azar (*n* = 50 kala-azar patients, unless stated otherwise)

Characteristics	N	%
*Health seeking behaviour and access to services*		
Type of health facility where care was first sought for fever		
Government PHC/ Government District hospital	6	12
Quack (unqualified practitioner)	18	36
Private qualified physician	19	38
Didn’t contact anyone (found first on active case search)	6	12
Type of first health facility contact in context of migration		
With migration	10	20
Without migration	40	80
Place of diagnosis of kala-azar		
District hospital	33	66
In village during active case search	15	30
Others (private facility)	2	4
Person who motivated to get tested for kala-azar		
ASHA (Accredited Social Health Activist)	34	78
ANM (Auxiliary Nursing Midwife)	13	26
Self	2	4
Another community member	1	2
Informed about single day treatment after diagnosis	41	82
Informed about monetary incentive of Rs 7100 (USD 100)	44	88
Received monetary incentive (*n* = 45)	36	80
Focal spray conducted within 2 weeks of fever onset	20	40
Quality of spray in preceding focal IRS of May 2017 (*n* = 42)		
All rooms and animal shelter(s) spayed	21	50
One or more rooms or animal shelter(s) missed	21	50
Health worker asked for history of fever among other family members	46	92
*Awareness*		
Awareness regarding mode of spread of Kala-azar		
Through sandfly bite	16	32
Through mosquito bite	12	24
Through dirty water	21	42
Directly from infected person	1	2
Awareness regarding duration for which walls should not be smeared or painted after IRS		
More than 90 days	0	0
60–90 days	12	24
Less than 60 days	13	26
Don’t know	25	50
Having awareness that animal shelter spraying is necessary for kala-azar prevention	31	62
*Risk factors for kala-azar*		
Illiteracy	43	86
Belonging to scheduled caste	46	92
Occupation – labourer	28	56
Migration	29	58
Poverty (Per capita income per month < Rs 1000)	43	86
Domestic animal ownership	16	32
Animal shelter location within house	7	14
Residing in thatched mud house without windows *(kutcha house)*	26	52
Sleeping in room with unplastered walls	45	90
Sleeping on floor	24	48
Non-use of mosquito net	40	80

The median total out-of-pocket medical expenditure was Rs 2000 (IQR 0–5000) with the highest expenditure of Rs 50,000 for the index case. Only in 12 of the 50 patients, the first health facility contact was with the government health system on their own or through active case search by frontline workers, with services availed completely free of cost (Table 2). Known risk factors for kala-azar such as illiteracy, poverty, belonging to socially disadvantaged community, migration, residing in *kutcha* houses and sleeping in rooms with unplastered walls and non-use of mosquito nets were present in majority of these cases (Table 2).

### The setting of seasonal migration

**Fig. 6:**
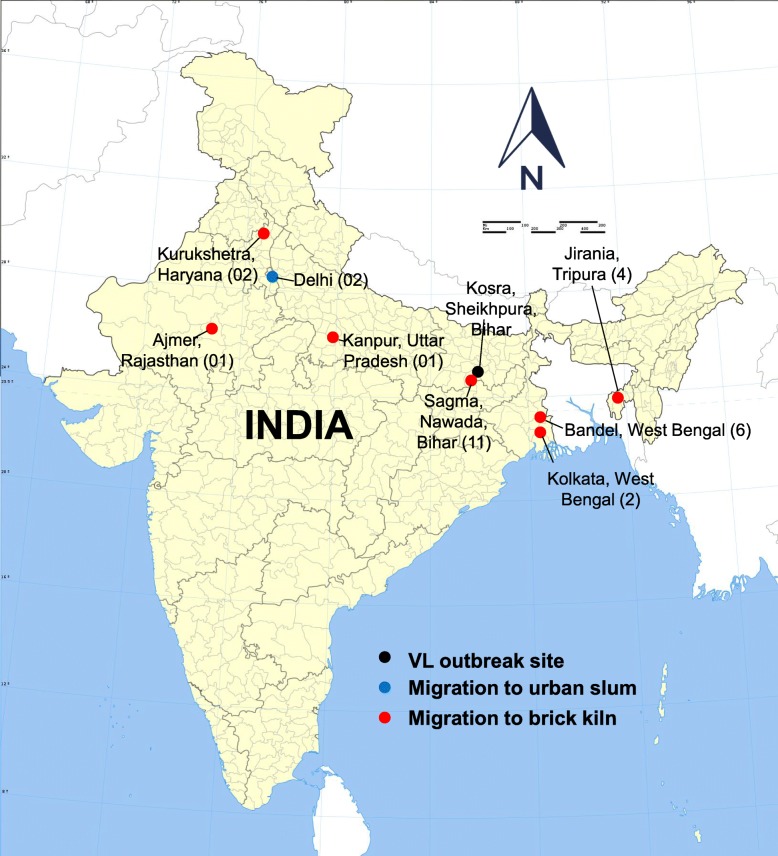
Category and location of migration sites of kala-azar cases (*n* = 29). *(Modified from source file -* https://commons.wikimedia.org/wiki/File:India_districts_map.svg*, Creative Commons Attribution-Share Alike 4.0 International license)*

Residents in the southern hamlet migrate seasonally for working in brick kilns located in northern and eastern part of India (Fig. 6). We observed lack of land ownership and poverty as the ‘push factors’ in prompting this migration [19, 20]. The migrants return to the village from the brick kilns in May and leave the village in October, thereby staying in the village during the pre-winter peak transmission period [16, 21].

Cases informed that contractors visit the southern hamlet of the village and lend sums of Rs 25,000–30,000 (USD 350–400) per labourer in advance to the families under an informal agreement to work in brick kilns. The sum received is used to pay back loans taken from local money lenders at high interest rates. Out of pocket medical expenditure are a major reason for taking these loans. This creates a self-perpetuating cycle of social exclusion, indebtedness, poverty and malnutrition for the migrants [19, 20].

### Verbal autopsy findings

Four children and one adult had died after 2–6 months of prolonged irregular fever, weakness, loss of appetite and pronounced abdominal swelling (only for children) in the most affected southern hamlet of the village. Blackish discoloration of skin was noted in the adult who died. Both genders were affected. The deaths happened between October 2016 – March 2017 before any diagnosis of kala-azar could be attempted. All five deaths could be epidemiologically linked to a family member or an immediate neighbour having been confirmed with kala-azar within 6 months of their death.

### Coverage of IRS and entomological study

Regular IRS round was done covering all the houses of the village –during 20–26 March 2017 and 26–31 August 2017. Focal spray was conducted in clusters of 70–200 houses around reported kala-azar cases in the southern hamlet during 15–17 May 2017, on 8 July 2017, 25–27 July 2017 and 20–22 December 2017.

Only half the dwellings of the kala-azar cases had been found to be fully sprayed during the most recent IRS conducted in May 2017 (Table 2). This was found to be inadequate as per norm of 80% coverage of household units [4, 22].

*P. argentipes* were collected from most of the households in the southern hamlet which had been sprayed thrice during the preceding four months. No other *phlebotomine* species were found. Six female *P. argentipes* were found to be fully gravid (Table 3). Mortality on mud and brick surfaces was below the recommended 80% level (Table 3) [22].

**Table 3:** Entomological findings in the southern hamlet of the affected village

Characteristics	n / total	%
*P. argentipes* density using mouth aspirator		
Households found positive for sandfly	28/ 34	82.4
Female percentage	14/ 37	37.8
*P. argentipes* density using CDC light trap		
Households found positive for sandfly	8/ 8	100
Female percentage	10/ 25	40.0
Cone bio-assay		
*Mud surface (n = 7 surfaces)*		
Knockdown	82/ 136	60.3
Mortality (24 h)	107/ 136	78.7
*Brick surface (n = 3 surfaces)*		
Knockdown	30/ 60	50.0
Mortality (24 h)	37/ 60	61.7

Bioassay control: 0% mortality

## Discussion

The intense spatial and temporal clustering of cases in the southern hamlet merits association with the index case. The long duration of untreated kala-azar in the index case would have sustained parasite transmission to sandflies present in the favourable eco-environmental setting of the village. Consequently, migrants residing in the village during the pre-winter transmission period would have been infected [21]. Subsequent development of fever in early part of 2017 nearly matches the average incubation period estimate of 4–6 months to 1 year [6, 23].

Clustering of kala-azar cases at meso-scale level has been reported in studies from Vaishali district, India and Dharan, Nepal [24, 25]. The present study uniquely reports an intense clustering even within a hamlet level at a scale of within 100 m. This is known to be partly due to the short flight range and enhanced transmission efficiency of the infected *P. argentipes* vector through biting persistence and multi-host feeding [26, 27].

All age-groups being similarly affected with kala-azar suggests an outbreak in non-endemic or low-endemic setting which is unlike an outbreak in endemic settings wherein older age groups are less affected as a result of immunity acquired from past infection [28]. Poverty, dietary changes and subsequent malnutrition associated with migration might have acted as risk factors for kala-azar infection, through weakening of host immunity [29–31]. A study in Bangladesh found low serum zinc and retinol levels as predisposing to conversion from asymptomatic infection to clinical case [32]. Zinc is essential for cell-mediated immunity which is responsible for resistance to *Leishmania donovani* infection [33, 34]. Therefore, host micronutrient status has been proposed as a potential risk factor for leishmania infection in Bihar [29].

### Role of seasonal migration

Majority of cases in the present outbreak developed symptoms while they were still working in the brick kilns located in different parts of the country and got treated on return to their village. Poor access to health care and low index of suspicion of kala-azar in these non-endemic areas and the low-endemic area back home resulted in patients spending considerable time and money without proper treatment. Migration was also suggested to play a role in kala-azar outbreaks in Dharan in Nepal, Varanasi in India and among Somali refugees and Kenyan shepherds in Africa [25, 35, 36]. However, in the present outbreak, migration to brick kilns doesn’t appear to have played a role in kala-azar transmission to the migrants per se. Rather it probably emerged as a risk-factor for development of kala-azar disease owing to poor nutrition and stress and also contributed to delayed diagnosis and treatment.

Finally, given the presence of *P. argentipes* vector over large areas of Indian sub-continent, there is hazard of establishment of transmission in far-flung non-endemic areas [21]. This could be possible if the brick kilns employing migrants from kala-azar endemic areas have presence of *P. argentipes* vector and are close to permanent human settlements located within the vector’s flight range [26]. Although high vector density at brick kilns could be less likely due to the unique requirement of damp, organic rich conditions for their oviposition and breeding [37], this needs entomological exploration at those brick kilns with seasonal migration from kala-azar endemic districts.

### Kala-azar transmission after outbreak response

Following the outbreak response in June 2017, approximately one kala-azar case was being reported per month (Fig. 5). Also, we found that 8 of the 352 rK39-seronegatives found during the outbreak response were subsequently diagnosed with kala-azar and were treated for it (Fig. 3). Most of these individuals would have either been incubating or would have been asymptomatic during the outbreak response. Further, with four additional kala-azar cases being reported from the village in 2019 with the most recent case in September 2019 i.e. more than twice the average incubation period after the outbreak response, it appears that kala-azar transmission couldn’t be completely interrupted in the village [23].

Dwellings of only half the kala-azar cases had been fully sprayed. Entomological findings pointed towards ineffective vector control during the outbreak. Resistance of vectors to Alpha-Cypermethrin insecticide could be ruled out as a reason for the bio-assay result since the affected area had been exposed to this insecticide only thrice prior to the assay. Cross-resistance with Dichlorodiphenyltrichloroethane (DDT) also could be ruled out as the village had no prior exposure to DDT for IRS (for either kala-azar or malaria) in the past 10 years. Thus, bioassay findings could be mainly explained by under-dosing of insecticide residue on walls in the three preceding IRS. Under-dosing could have been due to one or more of the following factors - patchy coverage of sprayable surfaces, less than recommended quantity of insecticide being used for suspension preparation, improper spray technique resulting in wider swath or faster spraying or due to less than recommended active ingredient concentration in the insecticide supplied [38]. We couldn’t assess all these factors in the present study.

In August 2018, the district programme was officially notified regarding 61 batches of Alpha-Cypermethrin wettable powder insecticide which had failed the recommended active ingredient specifications [39]. One of these failed batches of insecticide had been used in the affected village for the IRS rounds conducted in 2017. This could also help retrospectively explain the ineffective vector control leading to persistence of transmission in the present outbreak.

During the outbreak response, rk39-positive individuals providing history of fever without palpable spleen finding were not considered for treatment, as per programme guidelines [11]. Also, since fever is initially low-grade and intermittent in kala-azar, this symptom might not always be confirmed clinically. Spleen becomes palpable only after being enlarged twice to thrice [40]. Therefore, transmission potential of such cases should also be considered [41, 42]. Ultrasonographic detection of sub-palpable splenomegaly and bone marrow/intercostal splenic parasitology has been found to provide additional yield of kala-azar cases [43]. These should be explored, especially in outbreak settings. Findings of the present study could be generalizable to low-endemic and previously high-endemic areas for kala-azar in the Indian sub-continent.

### Limitations and sources of potential bias

Due to the insidious onset and prolonged fever seen in kala-azar, recall bias could have crept in while assessing duration of fever preceding kala-azar diagnosis.

## Conclusions

The interruption of transmission in outbreaks must rely on both complete case finding and effective vector control. This could be achieved through coverage of all sprayable surfaces during IRS and use of insecticide compliant with quality norms and plastering the walls of mud houses. Kala-azar, a neglected tropical disease, was found to disproportionately affect the poor and marginalized community.

This outbreak revealed the need to strengthen kala-azar elimination programme especially in low endemic and migratory settings so that future kala-azar outbreaks could be prevented and detected early. For example, establishment of routine framework for kala-azar outbreak response is needed owing to risk of outbreaks in low-endemic areas and areas which have hitherto achieved elimination targets [44].

There is scope for further reduction of delay between fever onset and diagnosis through sensitization of government and private practitioners, frontline health workers and active case detection, especially in migratory settings. Reduction of this delay has the potential to not only reduce out-of-pocket medical expenditure, but also to prevent future outbreaks and lead to dramatic reduction of kala-azar incidence in Bihar, India [45].

## Data Availability

All data related to the study belongs to the District Health Society- Sheikhpura, Government of Bihar, India. Data is not being publicly shared due to potential identifiability of study participants in a small community, even after anonymization. Those interested to obtain the data may contact the District Vector Borne Disease Control Office, Sheikhpura with a statement of purpose at vbdspura@gmail.com .

## Abbreviations

CDC: Centers for Disease Control and Prevention, Atlanta, USA
DDT: Dichlorodiphenyltrichloroethane
DVBDCO: District vector borne disease control officer
ICMR: Indian Council of Medical Research
IQR: Interquartile range
IRS: Indoor residual spraying
LAmB: Liposomal amphotericin B
NVBDCP: National vector borne disease control programme
RMRIMS: Rajendra Memorial Research Institute of Medical Sciences, Patna, India
VL: Visceral leishmaniasis
WHO: World Health Organization
